# Intracardiac metastasis from known cervical cancer: a case report and literature review

**DOI:** 10.1186/1477-7819-11-107

**Published:** 2013-05-23

**Authors:** Seung Won Byun, Sung Taek Park, Eun Young Ki, Hyun Song, Suk Hee Hong, Jong Sup Park

**Affiliations:** 1 Department of Obstetrics and Gynecology, Seoul St. Mary’s Hospital, The Catholic University of Seoul, 505 Banpo-Dong, Seocho-Gu, Seoul 137-040, Korea; 2 Department of Thoracic Surgery, Seoul St. Mary’s Hospital, The Catholic University of Seoul, 505 Banpo-Dong, Seocho-Gu, Seoul 137-040, Korea; 3 Department of Medical Oncology, Seoul St. Mary’s Hospital, The Catholic University of Seoul, 505 Banpo-Dong, Seocho-Gu, Seoul 137-040, Korea

**Keywords:** Cardiac carcinoma, Cervical cancer, Human papilloma virus, Metastasis, Pulmonary embolism, Recurrent

## Abstract

Cardiac metastasis from known cervical cancer is rare. Even through a routine check-up, this type of metastasis can present as pulmonary emboli. Suspicion of this diagnosis in an oncology patient with complicating pulmonary emboli but no evidence of deep vein thrombosis is important, especially in cervical cancer patients with extensive pelvic lymph node metastasis and vascular invasion of a primary tumor. Early recognition may aid in improving the prognosis. We present a case of intracardiac metastasis arising from a squamous carcinoma of the cervix in a patient with pulmonary tumor emboli and review other cases from the literature.

## Background

Metastatic disease of the heart is rare. The incidence quoted in the current literature, based on autopsy, is approximately 1.23% [1]. The low incidence of cardiac metastasis is classically attributed to a combination of factors: continuous myocardial contraction, metabolic particularities of striated cardiac muscle, rapid flow of blood through the heart and lymph flow away from the heart [2].

Classically, there are four pathways of cardiac involvement: (1) retrograde lymphatic spread, (2) direct extension from the adjacent viscera, (3) hematogenous spread, and (4) transvenous extension through the vena cava into the right side chambers.

Myocardial involvement is much less common. It is usually the result of hematogenous spread, and is associated with widespread disseminated disease. Intracavitary, endocardial, or valvular metastatic deposits, such as the one described in our case, occur in less than 6% of cases [3]. Of particular interest, approximately 80% of this type of metastasis occurs in the right chambers and only rarely in the left chambers. This is attributed to the filtering role of the pulmonary circulation and the slower flow in the right chambers [4]. Clinical diagnosis of cardiac metastases is difficult and may go unrecognized until autopsy.

We report a rare case of human papillomavirus (HPV)-detected metastatic intracardiac mass from known cervical cancer. The clinical, radiological and histological features are described. Simultaneously, the literature for all reports of these rare intracardiac metastases is reviewed.

## Case presentation

A 32-year-old woman diagnosed in 2009 with International Federation of Gynecology and Obstetrics (FIGO) stage IIA2 cervical cancer(squamous cell carcinoma), presented to our hospital emergency room with exertional dyspnea lasting four days. The patient had previously received multimodality treatment from a multidisciplinary oncology group, which, because the patient was young and wanted to preserve ovarian function, included the following treatment: radical hysterectomy along with ovarian transposition and pelvic and para-aortic lymph node dissection and concurrent chemoradiation with six cycles of weekly cisplatin, including tomotherapy in pelvic and para-aortic lymph node area.

**Figure 1:**
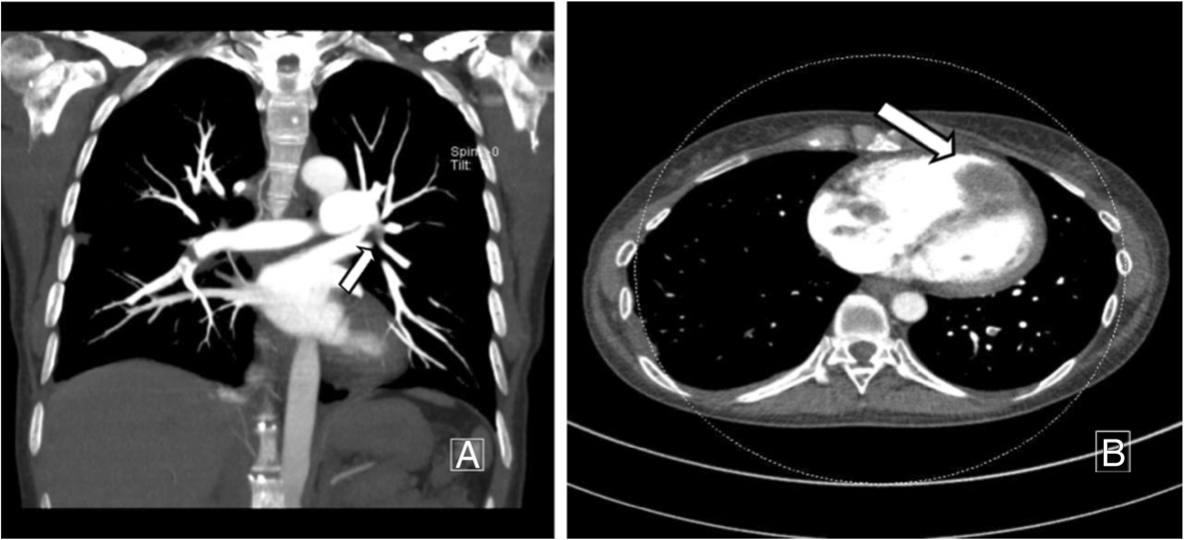
**Deep vein thrombosis three-dimensional CT (DVT 3D CT) angiography showing the cardiac mass and thromboemboli before the surgery.** (**A**) Maximum intensity projection (MIP) image shows pulmonary artery thromboembolism involving the right lower lobar artery and its segmental branches. (**B**) Computed tomography (CT) angiography of the pulmonary artery shows multiple large thrombi in the right atrium and right ventricle.

Thirteen months after completion of adjuvant treatment, 18-fludeoxyglucose positron emission tomography-computed tomography (18-FDG PET-CT) revealed no evidence of recurrence. However, just two months later, she was examined on admission with chest CT (Figure 1A), which reported a finding of thromboemboli in several segmental branches of the right middle and lower lobe pulmonary arteries and in left lower lobe posterior basal segment arteries. Therefore, she was anticoagulated with warfarin and enoxaparin, a low-molecular-weight heparin. However, unfortunately, the patient still showed clinical deterioration with increasing shortness of breath and developed signs of rightheart strain, suggestive of further episodes of pulmonary embolism.

For evaluation of the embolic source, we performed echocardiography and deep vein thrombosis three-dimensional CT (DVT 3D CT) angiography. The examinations revealed cardiac masses occupying the right ventricle and right atrium and no evidence of deep vein thrombus (Figure 1B).

**Figure 2:**
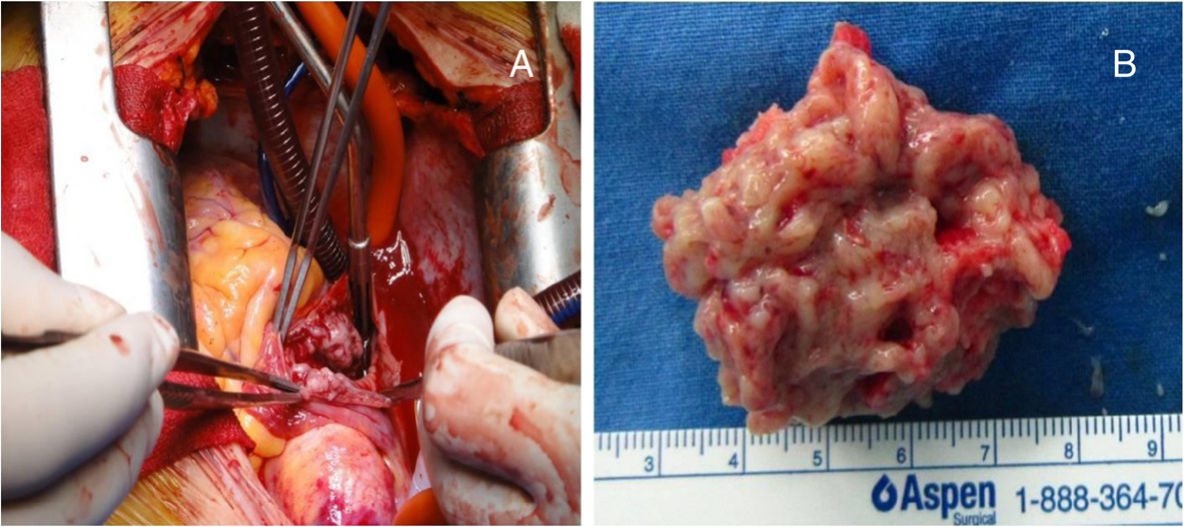
**Pictures showing open right atrium and gross finding of cardiac mass.** (**A**) The right atrium was open by cardiac incision. Cardiac masses invading the myocardium were seen. One of them protruded into the right ventricle through the tricuspid valve. However, the inferior vena cava was clear. Cardiac masses were also seen in the right ventricle and occupied 90% of the right ventricle lumen. All masses on the operation field were excised. (**B**) All materials from excised masses were collected. They were originally torn to pieces because the cardiac mass was so friable. Therefore, we fitted them together into one.

In view of the threatened outflow obstruction caused by the right ventricular mass, the patient underwent an open excision of the intracardiac mass (Figure 2A). At the opening of the right atrium, whitish fungating, friable masses were seen arising from the free wall of the right atrium, extending through the tricuspid valve into the right ventricle. The masses were also seen arising from the free wall of the right ventricle through the opened tricuspid valve and occupied 90% of the right ventricular lumen, nearly resulting in right ventricle outflow tract obstruction. The masses were partially sucked out through suction tubes. The remnants of the masses were excised and collected. They were originally torn to pieces because the cardiac mass was so friable. Therefore, we fitted them together into one (Figure 2B).

**Figure 3:**
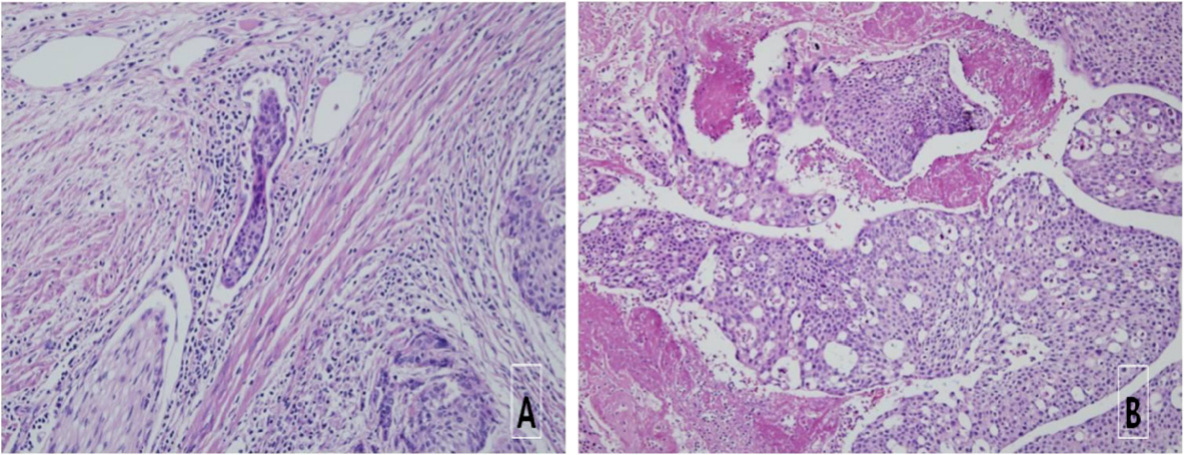
**Microscopic findings showing primary cervical cancer and cardiac metastasis.** (**A**) Microscopic findings of cervical squamous cell carcinoma (H&E, ×200 magnification). Vascular tumor emboli are observed adjacent to the tumor cell nest. (**B**) Microscopic finding of a right atrial mass. (H&E, ×100) Squamous cell carcinoma in the right atrum shows the same histology as the cervix. The tumor involves the myocardium.

The histopathology of the surgical specimen revealed this to be a cardiac metastasis from known cervical squamous cell carcinoma (Figure 3A). Additional HPV DNA genotyping of the metastatic lesion revealed HPV-16 consistent with the primary lesion. Cardiac metastasis should be the cause of the pulmonary embolic events and right heart failure that she had suffered.

After the patient’s condition was stable, she was transferred to a medical oncologist for recurrence management. She underwent carboplatin with paclitaxel chemotherapy for palliative treatment at the medical oncology department. On a follow-up chest CT and echocardiogram after three cycles of the chemotherapy, cardiac mass and pulmonary thromboemboli were no longer seen. The patient was supposed to undergo six more cycles of the chemotherapy. She tolerated the chemotherapy well, but during the last month of her life, the recurrent cancer mass surrounding the celiac axis caused extensive hepatobiliary obstruction. The cancer mass in her heart was still regressed. She died of cachexia one year and four weeks from diagnosis of cardiac metastasis.

## Conclusion

The clinical presentation may include nonspecific symptoms, such as chest pain, weight loss, malaise, or more characteristic symptoms, such as congestive cardiac failure secondary to intracardiac obstructions, valvular involvement, or pericardial effusions, arrhythmias due to involvement of the conduction system, or, as in our case, embolic events. Thus, metastases should be suspected in oncology patients if they develop inexplicable heart failure, neurological deficits, or recurrent pulmonary emboli, particularly when no peripheral source for the emboli can be identified.

Vascular invasion of primary cervical cancer is associated with hematogenous spread of cervical cancer (Figure 3B).

In our study, pathologic findings of primary cervical cancer showed vein and lymphatic invasion of squamous cell carcinoma with moderate differentiation. The authors suggest that the carcinoma of the cervix metastasized along the inferior vena cava to the heart, where it settled as an intracardiac obstructive mass. Pulmonary tumor emboli might decrease the right cardiac blood flow, resulting in a more hospitable environment for tumor cell adherence to the endocardial wall [5].

We searched PubMed between 1997 and 2011 and found nine articles, including the current case, which presented a clinical course and treatment of cardiac metastasis from known cervical cancer (Table 1) [6-12]. The average age of patients in these cases was 49.3 years old, the current case had the youngest patient who was 32 years old. While other cases didnot show any information about pathologic findings, our case showed identification of HPV DNA type 16 consistent with the primary tumor at the metastatic site. Yong Seok *et al*. suggested HPV status in the sentinel lymph node might be a prognostic factor in cervical cancer [13]. Yutaka *et al*. suggested that tumor-free, HPV DNA-positive metastatic sites should be monitored as being at high risk of relapse [14].

**Table 1:** Literature review of cardiac metastasis from cervical cancer cases

**Author(year)**	**Age**	**Stage**	**Type**	**Primary Tx**	**Interval to cardiac metastasis**	**Recurrence diagnosis modality**	**Pathology confirmation by**	**Recurrence Tx**	**Cause of death**	**Time to death from cardiac metastasis**	**Chief complaint for cardiac metastasis**	**Overall survival**
Ando *et al*. [6]	41	IIB	SCC	Op.	8M	MRI scan	Autopsy	CTx	RHF	5M	Dyspnea	13M
Lemus *et al*. [7]	53	Ib2	SCC	Op.	14M	MRI scan	Autopsy	CCRT	RHF	1M	Dyspnea	15M
Lemus *et al*. [7]	49	IVB	SCC	ERT	3M	MRI & CT scan	No autopsy	CCRT	RHF	7M	Dyspnea &tachycardia	13M
Inamura *et al*. [8]	58	IB1	SCC	CTx	44M	Echocardiogram and chest CT	Open excision	None	RHF	4M	Dyspnea &purpura of extremity	48M
Nakao *et al.*[9]	57	IIIB	SCC	CCRT	10M	Echocardiogram and chest CT	Open excision	None	RHF	2M	Mild chest pain & shortness of breath	12M
Borsaru *et al*. [10]	42	IVB	SCC	CCRT	6M	Echocardiogram and chest CT	Open excision	*	*	*	*	*
Kim *et al*. [11]	64	IB1	SCC	CCRT	5M	Echocardiogram, TEE and chest CT	Pericardiocentesis	CTx	RHF	7M	Dry cough&dyspnea	12M
Miller *et al*. [12]	48	Ib2	Adeno	CCRT	48M	MRI scan	Transesophageal echocardiography- guided biopsy	CTx/RT	RHF	8M	Chest pain	56M
Current study (2011)	32	IIA	SCC	Op.	15M	Echocardiogram and chest CT	Open excision	CTx	Cachexia	13M	Dyspnea &purpura of extremity	32M

*no available data. Adeno, adenocarcinoma; CCRT, concurrent chemoradiotherapy; CT, computed tomography; CTx, chemotherapy;ERT, external radiotherapy; M, months; MRI, magnetic resonance imaging; Op., operation; RHF, right heart failure; SCC, squamous cell carcinoma; TEE, transesophageal.Echocardiogram; Tx, therapy.

The role of HPV infection with a distant metastatic lesion is controversial. In a recent study, molecular analysis for HPV detection was performed in both uterine cervical cancer and right atrial cancer tissues. Both squamous cell carcinomas showed positivity for HPV, especially type 16. Based on this result, the tumor in the right atrium favors a metastatic tumor from the uterine cervix.

To our knowledge and based on our literature search, an HPV-detected intracardiac mass has never been reported to be associated with recurrence of known cervical cancer.

Cardiac metastasis from cervical cancer is very rare and difficult to diagnosis. It causes acute severe heart failure,a life-threatening condition for which there is currently no standard treatment. After our review of the clinical courses of cardiac metastasis from cervical cancer, we suggest that patients with a high risk of recurrence must be carefully evaluated using echocardiography for cardiac metastasis, even if there are no symptoms.

Further studies may reveal the risk and aggravating factors for cardiac metastasis from cervical cancer, and may provide additional evidence to support our suggestion.

## Consent

Written informed consent was obtained from the patient for publication of this case report and any accompanying images. A copy of the written consent is available for review by the Editor-in-Chief of this journal.

## Competing interests

The authors declare that they have no competing interests.

## Authors’ contribution

SWB contributed mainly to the design, literature review and the writing of the work. JSP, the corresponding author who provided the case, planned and approved the written work. STP and EYK helped correct the manuscript. HS performed the cardiac surgery and described the operative finding. SHH performed the palliative chemotherapy on the patient and edited the discussion. All authors read and approved the final manuscript.
